# *In Vivo* Evaluation of an Injectable Premixed Radiopaque Calcium Phosphate Cement

**DOI:** 10.1155/2011/232574

**Published:** 2011-05-31

**Authors:** Jonas Åberg, Eszter Pankotai, Gry Hulsart Billström, Miklós Weszl, Sune Larsson, Csaba Forster-Horváth, Zsombor Lacza, Håkan Engqvist

**Affiliations:** ^1^Division of Applied Materials Science, Department of Engineering Sciences, Uppsala University, 75237 Uppsala, Sweden; ^2^Department of Human Physiology and Clinical Experimental Research, Semmelweis University, Budapest H-1095, Hungary; ^3^Department of Orthopedics, Uppsala University, 75237 Uppsala, Sweden; ^4^Department of Orthopedics, Semmelweis University, Budapest H1113, Hungary

## Abstract

In this work a radiopaque premixed calcium phosphate cement (pCPC) has been developed and evaluated *in vivo*. Radiopacity was obtained by adding 0–40 % zirconia to the cement paste. The effects of zirconia on setting time, strength and radiopacity were evaluated. In the *in vivo* study a 2 by 3.5 mm cylindrical defect in a rat vertebrae was filled with either the pCPC, PMMA or bone chips. Nano-SPECT CT analysis was used to monitor osteoblast activity during bone regeneration. The study showed that by adding zirconia to the cement the setting time becomes longer and the compressive strength is reduced. All materials evaluated in the *in vivo* study filled the bone defect and there was a strong osteoblast activity at the injury site. In spite of the osteoblast activity, PMMA blocked bone healing and the bone chips group showed minimal new bone formation. At 12 weeks the pCPC was partially resorbed and replaced by new bone with good bone ingrowth. The radiopaque pCPC may be considered to be used for minimal invasive treatment of vertebral fractures since it has good handling, radiopacity and allows healing of cancellous bone in parallel with the resorption of the cement.

## 1. Introduction

Due to an aging population together with a lifestyle that requires less movement, many elderly today have weak bone structure. One complication with a weaker bone structure is an increased fracture risk, especially in the vertebras. Vertebral compression fractures are painful. In order to relief the pain the fracture can be stabilized using vertebroplasty [1]. Traditionally polymethylmethacrylate (PMMA) cement has been used in this procedure. However there are complications associated with the use of PMMA, such as cement leakage and increased risk of adjacent fractures [2, 3], so there is increased interest in finding alternative materials. Calcium phosphate cements (CPCs), which were first presented in the beginning of the 80s [4, 5], are alternatives to PMMA. Thanks to the chemical resemblance to bone the CPCs have good biocompatibility and are resorbed *in vivo* [6, 7]. CPCs consist of calcium phosphate salts, which upon mixing with water dissolve and then precipitate to form a solid body of either brushite or apatite depending on the pH of the solution [4]. There are already numerous commercial CPCs used in the clinical practice as bone void fillers [8]; however very few are intended for use in vertebroplasty. There are two major obstacles for the CPCs when to be used in spinal applications: handling and radiopacity. To obtain a CPC with sufficient working time, which can be injected through a G11 or G13 needle without filter pressing, is not an easy task, and various publications on injectability and setting regulators can be found [9–13]. Recently an innovative approach was taken where water was exchanged for glycerol as mixing liquid [14]. This gives the surgeon unlimited time for the injection, and the viscosity remains constant until the premixed calcium phosphate cement (pCPC) is in place and the cement comes in contact with water. The premixed concept has been shown to work on both brushite and apatite cements [14–16]. During injection into the vertebra it is important to be able to follow the cement with X-ray to avoid leakage. In the case of PMMA this is solved by adding either ZrO_2_ or BaSO_4 _ [17, 18]. A number of additives have been suggested to add radiopacity to CPCs, such as bismuth [19] and strontium [20]. However very little has been published describing CPC with ZrO_2_ and BaSO_4_, which are well documented for use in PMMA cements. 

The aim of this paper is to evaluate a premixed calcium phosphate cement containing ZrO_2_, regarding its setting time, compressive strength, and radiopacity as well as its *in vivo* behaviour in a vertebral rat model. 

## 2. Methods

### 2.1. Effects of ZrO_2_ Content

#### 2.1.1. Cement Preparation

The powder part of the cement consisted of *β*-tricalciumphosphate (*β*-TCP, Fluka), monocalcium phosphate monohydrate (MCPM, Scharlau), and zirconia (ZrO_2, _Sigma-Aldrich). MCPM and *β*-TCP were mixed in equimolar amounts. To this 0, 10, 20, 30, or 40% (w/w) ZrO_2_ was added. The powder was then mixed with glycerol (99%, Sigma-Aldrich). In all evaluated compositions the powder/liquid ratio was 4.2 g/mL. Before mixing, the *β*-TCP and ZrO_2 _were sterilized using heat (200°C during 2 hours [21]), and the MCPM was sterilized by soaking in 70% ethanol for >24 hours.

#### 2.1.2. Compressive Strength

After mixing, the paste was injected into cylindrical moulds *∅* 6 mm, height 12 mm open at both ends for compression strength testing and then immersed in 37°C phosphate buffered saline (PBS, pH 7.4, Sigma-Aldrich) to simulate physiological pH and start the setting reaction. After 24 hours the samples were removed from the mould, and a universal testing machine (Shimadzu AGS-H) was used with a cross-head speed of 1 mm/min; the maximum compressive stress was recorded for each sample. A thin plastic film was placed between the sample and the crosshead in order to reduce the effect of defects deriving from the mould. Six samples were tested per group.

#### 2.1.3. Setting Time

After mixing the cement paste was injected into 4 cylindrical moulds *∅* 6 mm, height 3 mm for setting time. To initiate the setting of the cement the moulds were immersed in 37°C PBS. The cement was considered to have set when the sample could support the 453.5 g Gillmore needle with a tip diameter of 1.06 mm without breaking. The four samples were tested consecutively with a 4-minute interval between each test. The mean between the time when the sample supported the weight and the previous time where the sample broke under the load was regarded as the setting time. Three measurements were made for each group.

#### 2.1.4. Radiopacity

For X-ray opacity measurements 1 mm thick samples were produced. The X-ray opacity was measured at 1 mAs, with 40 and 80 kV. A 1 mm sample of a PMMA-based cement (Vertebroplastic, DePuy, USA), which has barium sulphate as radiopacifier, was used as control along with an aluminium wedge 0.5–2.5 mm in 0.5 mm steps.

### 2.2. In Vivo

The cement used in the *in vivo* studies was prepared as described above containing 20% ZrO_2_. 1 mL syringes were prefilled with the premixed cement under aseptic conditions 4 days before the *in vivo* experiments. The syringes were stored at 8°C until surgery.

#### 2.2.1. Animal Surgery

Three-month old, 480–600 g male Wistar rats were anesthetized with halothane. The surgical area was washed with betadine three times, and a ligature was applied at the root of the tail to prevent bleeding. After removing the distal part of the tail a 2.0 mm diameter and 3.5 mm deep hole was drilled axially into the 4th or 5th tail vertebra. In order to stall the regeneration of bone a stainless steel wire was implanted into the hole. The wound was closed using a nonresorbable polypropylene suture. The localization of the wire was verified by X-ray. Twelve weeks later the animals were anesthetized again, and the implanted wire was removed. The hole was filled either with PMMA (Heraeus Palacos R) (*n* = 5), pCPC (*n* = 5), or with impacted human lyophyilized bone chips (*n* = 5), and the wound was closed. Bone regeneration was followed with nano-SPECT/CT. Twelve weeks later the animals were overanesthetized and sacrificed. The last two vertebrae (last operated vertebra plus one healthy vertebra) were fixed in 4% formaldehyde and analyzed with micro-CT and histology.

#### 2.2.2. Nano-SPECT/CT Analysis

Single isotope nano-SPECT/CT (Bioscan) imaging acquisitions were performed on 2-3 rats from each group in order to follow the integration of the bone substitutes and bone regeneration. Nano-SPECT/CT was carried out weekly for 6 weeks and once again on the 12th week. The rats were inoculated with 0.5 mL, 150 MBq of ^99m^Tc-methyl diphosphonate (^99m^Tc-MDP) through the tail vein under halothane anesthesia. Metastable technetium (^99m^Tc) is tagged onto a phosphonate compound such as MDP to generate ^99m^Tc-MDP, which selectively concentrates in osteoblasts. The selective accumulation of ^99m^Tc-MDP is ensured by both chemical adsorption onto the surface of the hydroxyapatite in bone and incorporation into the crystalline structure of hydroxyapatite [22]. Two hours later the animals were anesthetized with euthasol intraperitoneally, and 30-minute image acquisitions were performed. Regeneration activity was expressed as a percentage compared to the proximal healthy vertebra depending on isotope density.

#### 2.2.3. Micro-CT Analysis

The operated vertebrae were examined with microtomography (Skyscan 1172 X-ray microtomography Skyscan, Kontich, Belgium). Scans were carried out applying a 60 kV voltage and an Al-filter. Reconstruction was done with a modified Feldkamp algorithm using Skyscan Nrecon software. Microtomographical reconstruction was obtained by rotating the view through 180 degrees (rotation step 0.5 degrees). SkyScan CTvox (Kontich, Belgium) was used for the 3D visualization. 

#### 2.2.4. Histology

The formaldehyde-fixed 4th and 5th vertebrae were decalcified by immersing the samples in Biodec-R solution for 1 week. Five micron longitudinal sections were cut from the paraffin blocks and mounted on glass slides. Conventional hematoxylin-eosin (Merck & Co) staining was used to confirm the results of the micro-CT measurements.

## 3. Results

### 3.1. Evaluation of ZrO_2_ Content

#### 3.1.1. Compressive Strength and Setting Time

**Figure 1:**
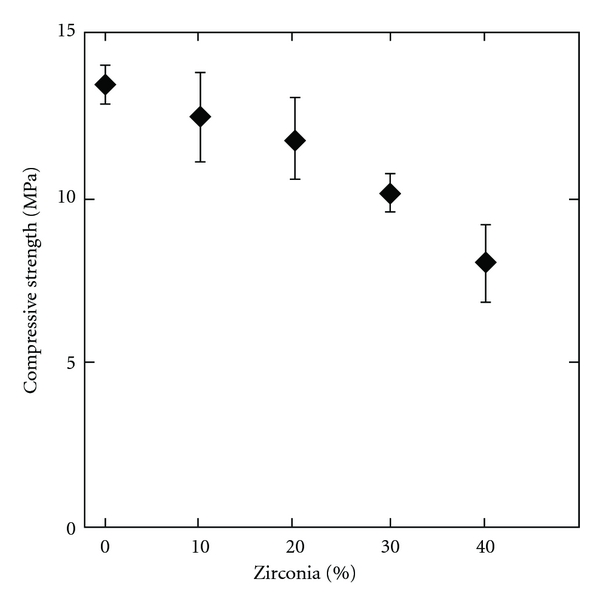
Diagram showing the influence of ZrO_2_ content (0 to 40%) on compressive strength of the pCPC after 24 hours incubation in PBS at 37°C. Error bars represent standard deviation, *n* = 6.

**Figure 2:**
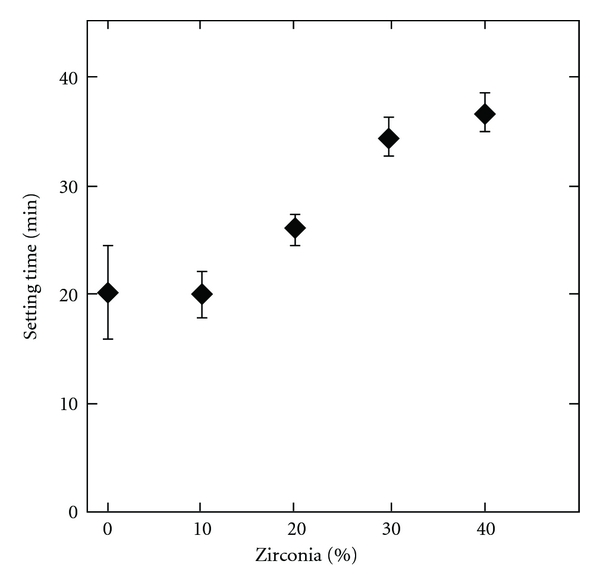
Diagram showing the influence of ZrO_2_ content (0 to 40%) on setting time of the pCPC. Error bars represent standard deviation, *n* = 3.

The compressive strength is reduced with an increasing content of ZrO_2_ as seen in Figure 1. When there is no ZrO_2_ in the cement the compressive strength is 13.5 (±0.6) MPa, and with 40% ZrO_2_ the compressive strength is lowered to 8.0 (±1.2) MPa. Values in parenthesis are the standard deviation. With 20% ZrO_2_ the strength is 11.8 (±1.2) MPa. An increasing amount of ZrO_2_ also increased the setting time as seen in Figure 2. 0% ZrO_2_ gives a setting time of 20 (±4) min, 20% ZrO_2_ has a setting time of 26 (±2) and the longest setting time was measured with 40% ZrO_2_, 37 (±2) min. 

#### 3.1.2. Radiopacity

**Figure 3:**
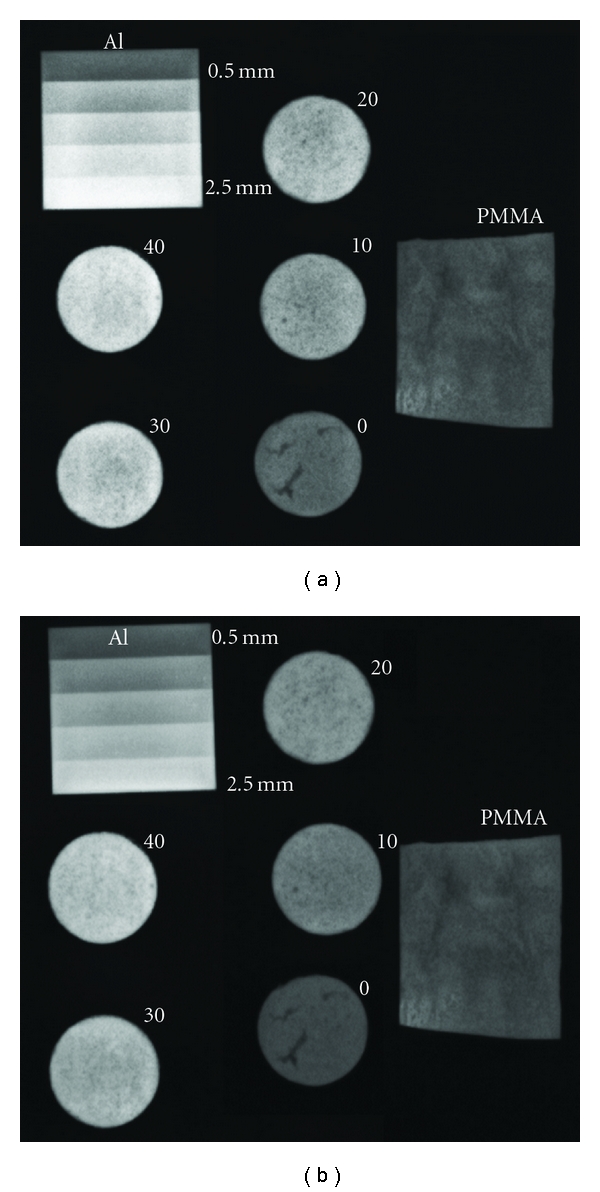
X-ray image comparing pCPC samples containing 0 to 40% ZrO_2_, with a commercial PMMA cement (Vertebroplastic) and an aluminium wedge with 0.5 to 2.5 mm in thickness. The X-ray opacity was measured at 1 mAs, with 40 kV (a) and 80 kV (b).

From the X-ray images in Figure 3 it could be concluded that 20% ZrO_2_ gave a sufficient radiopacity, comparable to 1–1.5 mm aluminium and Vertebroplastic at 40 kV and 80 kV. A good radio contrast was obtained *in vivo* with the pCPC, which can be observed in the micro-CT images in Figure 6. 

### 3.2. In Vivo

The animals tolerated all the implants well, there was no sign of wound infection or rejection. 

#### 3.2.1. Nano-SPECT/CT Analysis

**Figure 4:**
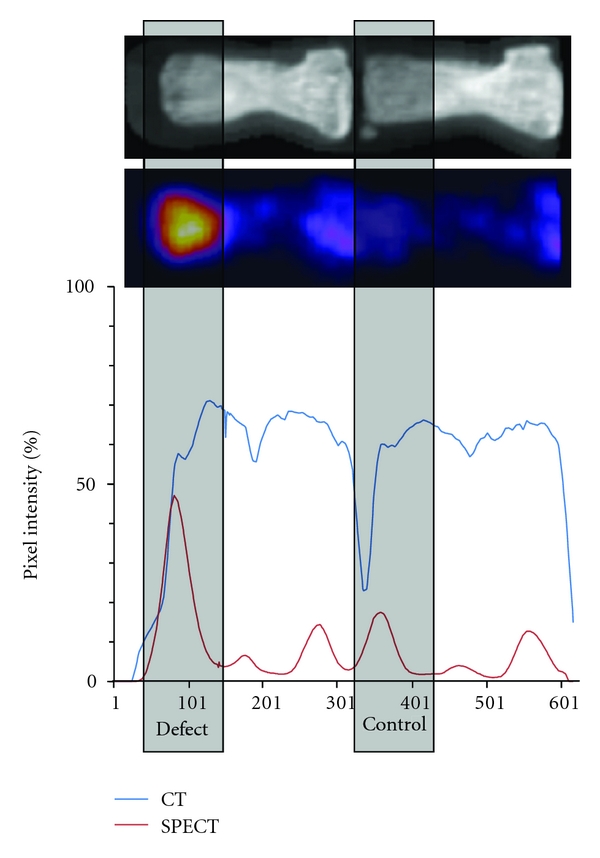
Comparison of intensities from CT (blue line) and nano-SPECT (red line) images. A representative image shows that the peak intensity of a vertebral epiphysis can be localized on the basis of the CT image and compared to the defect site. SPECT intensities reflect osteoblast activity. The defect site has a significantly higher osteoblast activity than the normal bone.

**Figure 5:**
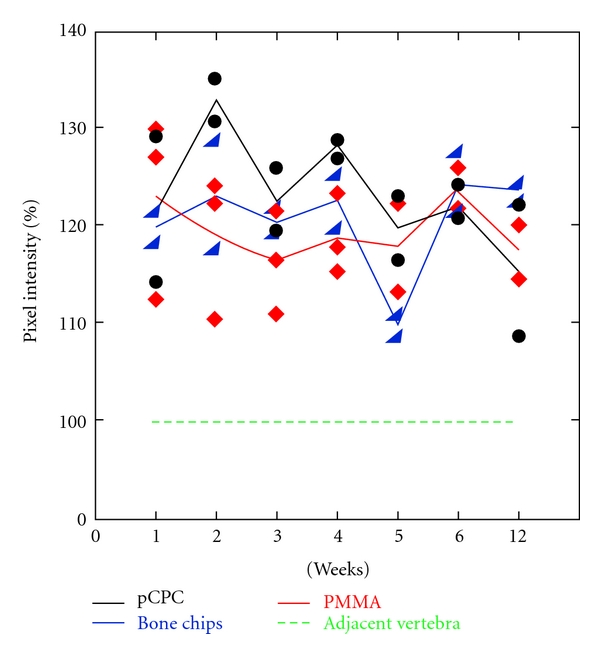
Diagram showing the osteoblast activity in the operated vertebra compared to the adjacent vertebra. The lines represent the mean value and the dots the individual animals value. The 12-week nano-SPECT/CT followup showed the highest osteoblast activity in the pCPC group during the first 5 weeks. Although the differences between the three groups level off after the 5th week, pixel intensity stays higher in all three groups compared to the neighbouring healthy vertebra.

The osteoblast activity was monitored during 12 weeks after implantation (Figure 5). The activity was calculated as the percentage of the next healthy vertebra. The CT graph (blue line in Figure 4) follows the structure of the two vertebrae, while the pixel intensity is much higher in the operated vertebra compared to the healthy control vertebra when looking at the red line in Figure 4. The same trend was observed in all animals. In all three groups the values are higher than those of the normal bone as seen in Figure 5. The osteoblast activity in this cavitary defect is higher than the normal bone and remains high during the first 5 weeks after implantation. The overall activity is comparable among the groups, with a slight trend of pCPC for an even higher activity, especially at week 2, while a slight decrease can be observed in the bone chips and the PMMA groups. The osteoblast activity did not return to normal levels (unit 100 in the graph) in any animals in any group during the study.

#### 3.2.2. Micro-CT Analysis

**Figure 6:**
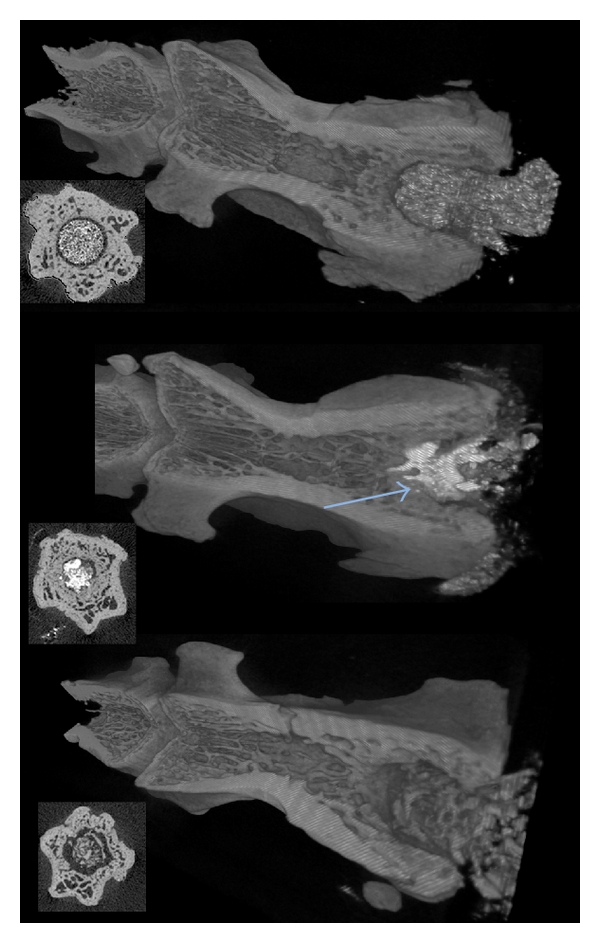
The representative micro-CT images after 12 weeks of the operated vertebra. PMMA (upper image) is compacted in the hole, but no bone formation was observed; a radiolucent area is seen between the PMMA and the bone. The pCPC (middle image) is slowly resorbed, and new bone is formed in connection with the pCPC (arrow). There was some bone formation around the bone chips, however considerable radiolucent areas can also be observed.

In Figure 6 representative 3D renderings of the filled defect along with cross-sections are presented. As seen in the figure, in the vertebrae filled with PMMA (upper), the defect was completely filled with PMMA and no bone formation was observed. The PMMA was demarcated from the bone. This result was consistent in all animals in the PMMA group. 

**Figure 7:**
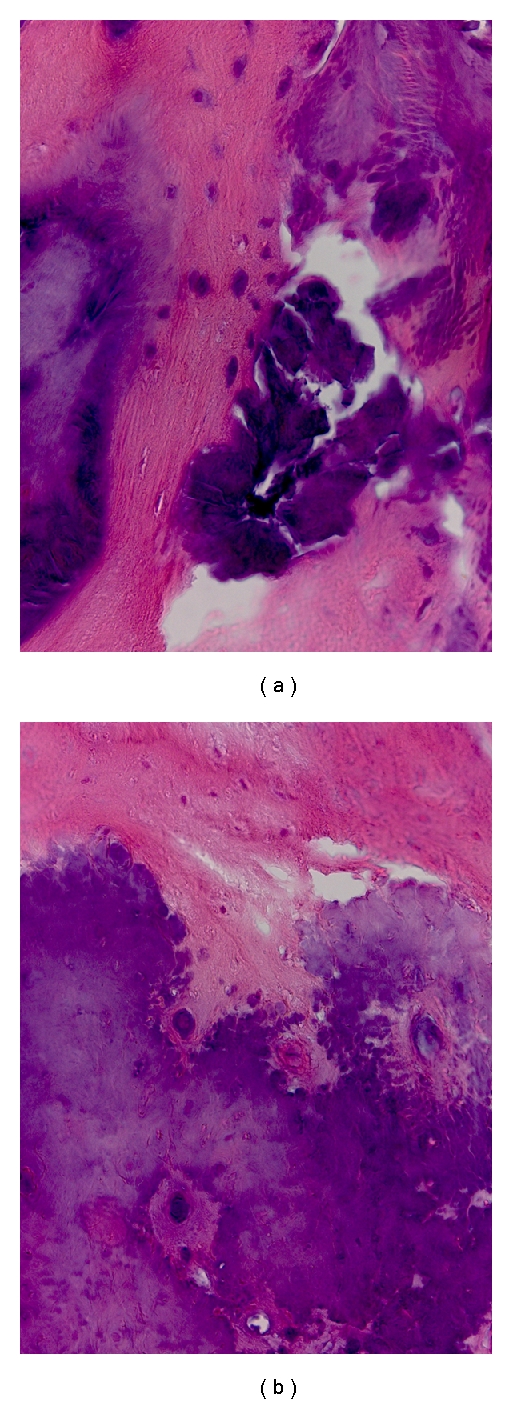
Histological signs of remodelling. Hematoxyline-Eosine stained sections. (Magnification: 40x). (a) shows the injected pCPC in contact with newly formed bone. (b) shows an implanted bone chip. Newly formed bone is seen in contact with the slowly remodelling calcified bone fragment.

In the vertebrae filled with bone chips no or slow resorption could be observed, and limited bone healing. There was no bony connection between the bone chips and the new bone as seen in the cross-section CT image in Figure 6 (bottom). There was some bone formation; however the bone chips are still demarcated from the bone. In the histology image in Figure 7(b) bone chips are seen to have direct contact with newly formed bone. 

In the case of pCPC in the vertebrae (middle image in Figure 6) new bone formation can be observed (blue arrow). The pCPC has been partially resorbed and replaced with bone. However the ZrO_2_ present in the pCPC for radiopacity is not resorbed, and thus residues of the cement can be observed in the newly formed bone. Histology results, (Figure 7(a)) confirmed the nanoSPECT and CT results for the pCPC. Direct contact with newly formed bone and no immunologic reaction was observed.

## 4. Discussion

The premixed cement presented here contains ZrO_2_ to obtain radiopacity. An increasing amount of ZrO_2_ in the cement pastes will decrease the amount of reactive material (MCPM and *β*-TCP). When the cement comes in contact with water the reactive material will dissolve and monetite crystals start to form giving the paste its strength. Accordingly the compressive strength was reduced from 13.5 (±0.6) to 8.0 (±1.2) MPa with an increase of ZrO_2_ from 0 to 40% ZrO_2_ (Figure 1). In relation to this the setting time increased with an increasing amount of ZrO_2_. 0% ZrO_2_ gave a setting time of 20 (±4) minutes compared to 37 (±2) minutes with 40% ZrO_2_ (Figure 2). The same trend was seen in a previous publication on premixed monetite cement [16] where high strength samples also showed shorter setting time. Since the method used for testing the setting time measures how fast the cement obtains a certain strength this relation is expected. As the ZrO_2_ content increases it was noted during the experiments that the viscosity of the cement decreases. Since the ZrO_2_ has higher density than the *β*-TCP and MCPM more ZrO_2_ implies that the total powder volume will be smaller when using the same weight of powder. With less powder volume the glycerol will more easily encase the powder particles thus reducing the viscosity. With 20% ZrO_2_ the compressive strength was 11.8 (±1.2) MPa and the setting time 26 (±2) minutes, which are acceptable values for certain vertebral fractures, and this also gave a good radiopacity. Therefore this formulation was chosen for the *in vivo *studies.

As seen in the results from the nano-SPECT (Figure 5) the osteoblast activity in this cavitary defect is higher than the normal bone and remains high during the 12-week duration of the study. This indicates that bone formation was ongoing throughout the study. Although there were too few animals in the study to do statistical analysis on the results from the nano-SPECT a slight trend could be observed indicating that the pCPC has a positive effect on the osteoblast activity compared to the other groups. It is most clear in weeks 2, 3, and 4. The monetite formed when pCPC sets is chemically less stable than apatite under *in vivo *conditions where the pH is ~7.4, thus it can be resorbed faster, than, for example, apatite forming CPCs and bone grafts [23]. When the pCPC is resorbed, calcium and phosphate ions are released which stimulate the osteoblasts [24]. This could explain why the osteoblast activity seems to be slightly higher in the pCPC animals.

Looking at the CT results the PMMA group present no surprises. The PMMA fills the defect, and it cannot be resorbed, thus any new bone formation into the cavity is blocked. The results from the bone chips on the other hand show new bone formation into the cavity; however the bone chips do not show signs of bony reconsolidation, and it is clearly demarcated from the host bone. This is probably due to the highly compacted structure of the bone chips, so their osteoinductive potential can only be seen on the contact surface, and penetration of the graft by the host tissue is limited. 

In the pCPC group new bone formation was observed and there is connection between the new bone and the material. This indicates that the pCPC has osteoconductive properties, which has been shown for monetite in previous publications [25, 26]. The resorption of pCPC and new bone formation, that is, the remodelling, seems to be in balance in this model. The ZrO_2_ is not resorbed; however the CT results indicate that good bone integration was achieved around the ZrO_2_. Previous studies where ZrO_2_ was used in contact with bone also report good bone-implant contact [27]. In future studies the bone integration of ZrO_2_ will be evaluated more thoroughly.

## 5. Conclusions

The premixed calcium phosphate cement containing 20% ZrO_2_ was found to have excellent handling, good radiopacity, and sufficient strength and setting time.

All materials evaluated in the study filled the bone cavity, and in each case there was a strong osteoblast activity at the injury site. However, in spite of the osteoblast activity, PMMA blocked bone healing, and even bone chips seemed to be less effective than pCPC. The pCPC was partially resorbed and replaced by new bone.
